# Transcriptomic Analysis of Circulating Leukocytes Reveals Novel Aspects of the Host Systemic Inflammatory Response to Sheep Scab Mites

**DOI:** 10.1371/journal.pone.0042778

**Published:** 2012-08-03

**Authors:** Stewart T. G. Burgess, Andrew Greer, David Frew, Beth Wells, Edward J. Marr, Alasdair J. Nisbet, John F. Huntley

**Affiliations:** 1 Moredun Research Institute, Pentlands Science Park, Bush Loan, Edinburgh, Midlothian, Scotland, United Kingdom; 2 Faculty of Agriculture and Life Sciences, Lincoln University, Lincoln, Canterbury, New Zealand; Kyushu Institute of Technology, Japan

## Abstract

Infestation of ovine skin with the ectoparasitic mite *Psoroptes ovis* results in the development of a rapid cutaneous inflammatory response, leading to the crusted skin lesions characteristic of sheep scab. To facilitate the identification of novel diagnostic and therapeutic targets, a better understanding of the host-parasite relationship in sheep scab is essential. Although our knowledge of the host's local cutaneous inflammatory response to sheep scab has increased in recent years, we still know relatively little about the mechanisms of this response at the systemic level. This study used a combined network and pathway analysis of the *in vivo* transcriptomic response of circulating leukocytes to infestation with *P. ovis*, during a 6 week period. Network graph analysis identified six temporally-associated gene clusters, which separated into two distinct sub-networks within the graph, representing those genes either up or down-regulated during the time course. Functional and pathway analysis of these clusters identified novel insights into the host systemic response to *P. ovis* infestation, including roles for the complement system, clotting cascade and fibrinolysis. These analyses also highlighted potential mechanisms by which the systemic immune response to sheep scab can influence local tissue responses *via* enhanced leukocyte activation and extravasation. By analysing the transcriptomic responses of circulating leukocytes in sheep following infestation with *P. ovis*, this study has provided key insights into the inflammatory response to infestation and has also demonstrated the utility of these cells as a proxy of events occurring at local tissue sites, providing insight into the mechanisms by which a local allergen-induced inflammatory response may be controlled.

## Introduction

Sheep scab, or ovine psoroptic mange, caused by infestation of sheep skin with the mite *Psoroptes ovis* is highly contagious, causing intense pruritus and irritation, resulting in a disease of major welfare concern [1]. Current disease control strategies rely on the use of acaricidal dips and endectocides but the emerging issues of biological residues, eco-toxicity and development of acaricide resistance have raised concerns regarding the sustainability of this strategy, highlighting interest in the development of alternative control methods [2]. To develop alternative methods of control a deeper understanding of both the parasite and its interaction with the host are essential. Although the basic biology of the host∶parasite interaction is well understood, there is a paucity of information about the mechanisms underlying the host response, in particular at the systemic level. Sheep scab infestation is characterised by three distinct phases, “early”, “late” and a subsequent “decline” phase [3]. During the early phase, the host's skin becomes reddened and inflamed within minutes of infestation and within 24 hours an epidermal influx of neutrophils (representing the majority of infiltrating cells) and eosinophils can be observed, followed by blister formation and a pronounced serous fluid exudate and dermal oedema [4]. Increases in dermal mast cell numbers occur by 96 hours post-infestation, and *P. ovis*-specific IgE is detectable within one week post-infestation [3]. These histopathological and serological changes suggest an immediate-type hypersensitivity reaction may be involved in lesion development [5]. However, this does not fully explain the initial pro-inflammatory response, as this occurs in mite-naïve sheep which lack *P. ovis*-specific IgE [3]. We have shown previously that, within 24 hours of a primary infestation, the expression of genes encoding pro-inflammatory/pro-allergic mediators [including colony stimulating factor 2 (*CSF2*), interleukin-1A ( *IL1A*), *IL1B*, *IL4*, *IL8*, *IL6* and *IL13*] is increased in sheep skin [6]. Expression of these cytokines is likely to be caused by keratinocyte activation, as *P. ovis* is a non-burrowing mite and these cells form the first point of contact between host and parasite [6]. This early stage can last from 2–3 weeks and clinical symptoms may not be observed without close examination during this time [3]. During this period the mite population increases and the lesion begins to expand, eventually spreading across the body [3]. In response to the intense itching and increasing mite population, it is during this late phase that the clinical signs of disease become most apparent. In experimentally infested animals, this late phase continues for a further 2–4 weeks until adaptive host immune responses begin to affect mite numbers [3], [4]. Components of the pro-inflammatory response to pathogens have been identified within circulating blood cells in humans and these have been classified as the ‘systemic inflammatory response’ [7], [8]. Currently little is known about the role of systemic inflammation in the development of ovine psoroptic mange and analysis of components of the systemic inflammatory response will aid understanding of the mechanisms behind the host response to infestation. Several circulating leukocyte populations are present in sheep blood, including neutrophils (40–60%), eosinophils (1–4%), basophils (0.4–1%), lymphocytes (25–35%) and monocytes (4–6%) [9]. The activity of circulating leukocytes contributes to the levels of cytokines and other pro-inflammatory markers, both systemically and at local sites of inflammation [10]. As such these cells are able to influence the course and nature of an inflammatory response occurring at local tissue sites [10]. It has been proposed that, as circulating leukocytes are able to interact and communicate with every tissue in the body, they can act as a ‘sentinel tissue’ reflecting disease progression at local sites of infection [11]. Depending on the specific pathogen/parasite, the RNA expression profiles of these cells may possess a disease-specific signature, reflecting the gene networks and signalling pathways involved in the host response [12]. We described previously a transcriptomic analysis of the localised host skin response to infestation with *P. ovis*, elucidating the processes leading to the development of a rapid and profound cutaneous inflammatory response [6]. Here, to gain a greater understanding of the systemic host response, we investigated gene expression in circulating leukocytes, providing a series of “snap-shots” of the transcriptomic response in the host vasculature over the progression of an infestation. The transcriptomic profiles of circulating leukocytes were interrogated using a gene expression microarray and a combined clustering, network and pathway mapping approach enabling the identification of key signalling events in the host systemic response to *P. ovis*.

## Materials and Methods

### Animal study

Ethical approval for this study was obtained from the Moredun Research Institute Experiments Committee and animals were monitored daily in accordance with guidelines agreed with the UK Home Office. *P. ovis* mites (a mixed population consisting of adults, nymphs and larvae) were harvested from infested donor animals maintained at the Moredun Research Institute as described previously [6]. Scotch mule lambs (1–2 years old, n = 6) with no previous exposure to *P. ovis* were maintained at the Moredun Research Institute. Prior to infestation with *P. ovis* a blood sample (9 ml) was removed from each animal by venipuncture into a Bio-One 9 ml EDTA K3 Vacuette blood tube (Greiner, UK) and processed immediately as described below for the isolation of leukocytes and subsequent RNA extraction. These samples represented the baseline (Time = 0) for each animal. Approximately 20–50 mites were placed directly onto the skin at the withers of each animal and infestations allowed to proceed for 6 weeks. During this period blood samples were taken weekly and processed as described for the baseline samples. Lesion area measurements were obtained for all animals during the 6 week (42 day) period of infestation by measuring the length and breadth of each lesion.

### Isolation of leukocytes from whole blood

The analysis of gene expression in whole blood samples is hampered by the presence of large quantities of globin mRNA which can represent up to 70% of the transcript population, thus limiting the ability to accurately detect genes expressed at low levels [13]. In order to reduce the issue of globin mRNA transcript contamination we used a pre-filtration protocol to isolate leukocytes from whole blood samples prior to cell lysis and subsequent RNA extraction. Briefly, leukocytes were captured by passing the whole blood samples, less than 1 hour after removal, through an Ambion LeukoLOCK filter (Ambion, UK) under vacuum pressure. The filter was then flushed with 3 ml phosphate buffered saline (PBS) to remove residual erythrocytes and then 3 ml of RNALater (Ambion, UK) was passed into the filter to preserve RNA signatures before storage at −80°C, prior to RNA extraction.

### RNA extraction

RNA was extracted from the separated leukocyte samples following the manufacturer's protocol (Ambion). Briefly, the filter was thawed on ice and residual RNALater flushed out using an empty 5 ml syringe. The leukocytes on the filter were lysed by flushing with 2.5 ml of Lysis/Binding buffer and the flow through (lysis extract) collected in an RNase free tube. Residual proteins in the eluate were degraded using a brief (5 mins) Proteinase K digestion and RNA was captured using RNA-binding beads following the manufacturer's protocol. Residual DNA was removed following DNase I treatment (10 mins, TURBO DNase, Ambion, UK) and purified RNA was eluted into elution solution. RNA sample quality was assessed on an Agilent Bioanalyser (Agilent, UK), an RNA Integrity Number (RIN) obtained for each sample and RNA yield was assessed on a ND-1000 Nanodrop spectrophotometer (Thermo Scientific, UK). RNA samples with a RIN>7.5 were considered to be of acceptable quality for downstream microarray processing [14].

### Microarray study

As no suitable ovine microarray platform was available at the start of this study transcriptomic analysis was performed with the Agilent bovine gene expression microarray (43,803 probes representing 21,520 bovine genes) in a 4×44 K slide format. Sheep cDNA samples derived from each time point were randomly assigned to the arrays within a slide, with each sample (time point) for each individual animal hybridised onto the arrays on a single slide to limit technical variation. One array was employed for each of 4 time points [non-infected (time = 0), 1 week (7 days), 3 weeks (21 days) and 6 weeks (42 days) post-infestation] per animal. Six animals were used in the study, giving a total of 24 arrays. The Agilent One-Colour gene-expression workflow (Cy3 dye, Quick Amp Labelling Protocol, Agilent, UK) was used to amplify and process RNA samples, following the manufacturer's protocols. Briefly 800 ng total RNA was used for the generation of fluorescently labelled (Cy3 dye) complementary RNA (cRNA), using T7 RNA polymerase. Microarrays were hybridised at 65°C for 17 hours in an Agilent Microarray Hybridisation Oven (Agilent, UK), and scanned for Cy3 dye intensity on an Agilent Microarray Scanner (Agilent, UK) at the manufacturer's recommended settings. Microarray signal data were extracted using Agilent Feature Extraction software version 9.5.3 (Agilent, UK). To enable inter-array comparisons, raw data for each array were normalised to the 75^th^ percentile of all non-control probes within the Genespring GX 11.0 software package (Agilent, UK) prior to log transformation. Further downstream filtering of the normalised array data was performed in Genespring GX 11.0.

### Statistical analysis of microarray data

Differential gene expression across the time course of infestation was determined using a one way-analysis of variance (ANOVA) with a Student-Newman-Keuls (SNK) post-hoc test in Genespring GX 11.0 (Agilent Technologies, UK) comparing each time point, (time = 0, 1, 3 and 6 weeks post-infestation) across all animals. Multiple test correction was performed using the Benjamini & Hochberg False Discovery Rate (FDR) procedure with an FDR corrected p-value cut-off set at ≤0.05 and a fold change cut-off of ≥1.8 [15].

### Network graph analysis and temporal clustering of gene expression data

Normalised, log-transformed gene expression data along with unique identifiers and all available annotation for the significantly differentially expressed transcripts with a fold change ≥1.8 were imported into the network visualisation and analysis tool BioLayout Express 3D [16]. The software generated an “all sample *vs* all sample” Pearson correlation matrix based on the expression profiles of the selected probes. Probes with a Pearson correlation co-efficient >0.7 were stored and network graphs were generated at selected cut-off values above this threshold. The graphs consisted of nodes, representing the individual probes (genes), connected by edges representing expression correlations above the selected Pearson threshold. Using the Markov Clustering Algorithm (MCL) network graphs were clustered according to connectivity between individual nodes, producing groups of probes with similar expression profiles across the time course of infestation [16], [17]. In order to provide a metric for statistical significance, BioLayout Express 3D used a two-sided Fisher's exact test and a Bonferroni correction for multiple testing [16].

### Pathway and gene network analysis

Data were analysed using Ingenuity Pathway Analysis (IPA, Ingenuity Systems Inc, USA). Gene clusters identified from the network graph and clustering analysis were uploaded as the input data set into IPA. Each gene identifier was mapped to its corresponding gene object in Ingenuity's Knowledge Base. Gene networks were then algorithmically generated based on their connectivity and assigned a score indicating how relevant the network was to the genes in the input dataset. Networks were analysed to identify the biological and/or disease functions most significant to the genes in that network. Canonical pathway analysis identified the biological pathways of highest significance to the input data sets. The significance of the association between the data set and the canonical pathway was determined based on two parameters: (1) A ratio of the number of genes from the dataset that mapped to the pathway divided by the total number of genes that mapped to the canonical pathway and (2) a p-value, calculated using a Fisher's exact test, determining the probability that the association between the genes in the data set and the canonical pathway was due to chance alone.

### qPCR validation

Quantitative real-time PCR (qPCR) was used to verify differential expression of 10 selected genes from the final list of differentially expressed transcripts. Briefly, RNA from sheep blood samples, prepared as described above, was reverse transcribed into cDNA using anchored Oligo(dT)_23_ primers (Sigma, UK) and Superscript II reverse transcriptase (Invitrogen, UK) following the manufacturers' instructions. TaqMan qPCR was used to measure relative transcript levels using pre-validated “assay-on-demand” specific primer and probe sets (Applied Biosystems, UK) for the following genes: *SPP1* (Assay ID: Bt03213107_m1); *ALAS2* (Bt03221878_m1); *ALOX15* (Bt03214775_m1); *IGFBP4* (Bt03259499_m1); *MPO* (Bt03269033_m1); *C4BPB* (Bt03237460); *CTTN* (Bt03240896_m1); *PDLIM1* (Bt03222239_m1); *PLAU* (Bt03212959_m1) and *ANG* (Bt03279285_m1). At the time of validation ovine gene sequences were not available for the selected genes, so the homologous bovine gene sequences were used for primer and probe set selection and all values were normalised to the transcript levels of the bovine glyceraldehyde-3-phosphate dehydrogenase (*GAPDH*) internal control gene [Bt03210913_g1 (Applied Biosystems, UK)]. All sample analyses were performed in triplicate and the 2-ΔΔCt method was used to assess relative gene expression differences between the respective time points post-infestation (1, 3 and 6 weeks) for each animal compared to the baseline (t = 0) sample for each animal [18], [19]. Transcript levels, measured in this way were then normalised using the *GAPDH* internal control and expressed as fold change (FC) compared to the control (t = 0) reference sample. Genes for validation were selected to represent a range of the clusters identified in the network graph analysis and to provide a range of genes differentially expressed across different time points. The expression profiles of the selected genes were then compared to the corresponding microarray probe data.

## Results

### Sheep scab lesion development during infestation

Following infestation with *P. ovis*, lesion size (n = 6 sheep) was assessed weekly. Mean lesion areas (± SEM) were as follows: Time 0 = no lesions; one week post-infestation (1 wpi) = 12 cm^2^ (±3 cm^2^); 2 wpi = 79 cm^2^ (±26 cm^2^); 3 wpi = 176 cm^2^ (±59 cm^2^); 4 wpi = 347 cm^2^ (±148 cm^2^); 5 wpi = 772 cm^2^ (±429 cm^2^); 6 wpi = 1125 cm^2^ (±631 cm^2^). This represented an approximate doubling of lesion size each week for all animals and a linear increase over the 6 week infestation period.

### Isolation of RNA from ovine circulating leukocytes

Total RNA from ovine circulating leukocytes isolated from whole blood samples was successfully extracted from all samples with a mean RIN value of 9.4 (Min = 9, Max = 9.6) indicating high quality, purified RNA with little observable degradation. The mean yield of total RNA across all leukocyte samples (n = 24) used in the study was 36 µg (±1.67 µg) from a 9 ml whole blood sample.

### Microarray data processing

To ensure quality and consistency of the sample-labelling process and array hybridizations, control information was collated from all arrays. Quality control data were found to be consistent with the manufacturer's (Agilent) recommendations. The performance of the array hybridizations was further assessed through scatter plots (data not shown) comparing each array with every other generated. This confirmed linear distributions between arrays, showing a dynamic uninterrupted range of expression values from low to high signals. Box and whisker visualizations confirmed the data had comparable distributions and were of sufficient quality for further analysis (data not shown). Following data normalisation and log transformation, invariant transcripts, whose presence could contribute to multiple testing errors in the subsequent statistical analysis, were removed. Downstream data filtering of the array dataset (21,520 probes) was performed and probes with a “present” or “marginal” flag call in 100% of the samples at any one of the 4 time points were considered reliable for further analysis, resulting in a final list of 14,174 probes for the differential expression analysis.

### Determination of differentially expressed transcripts

**Figure 1:**
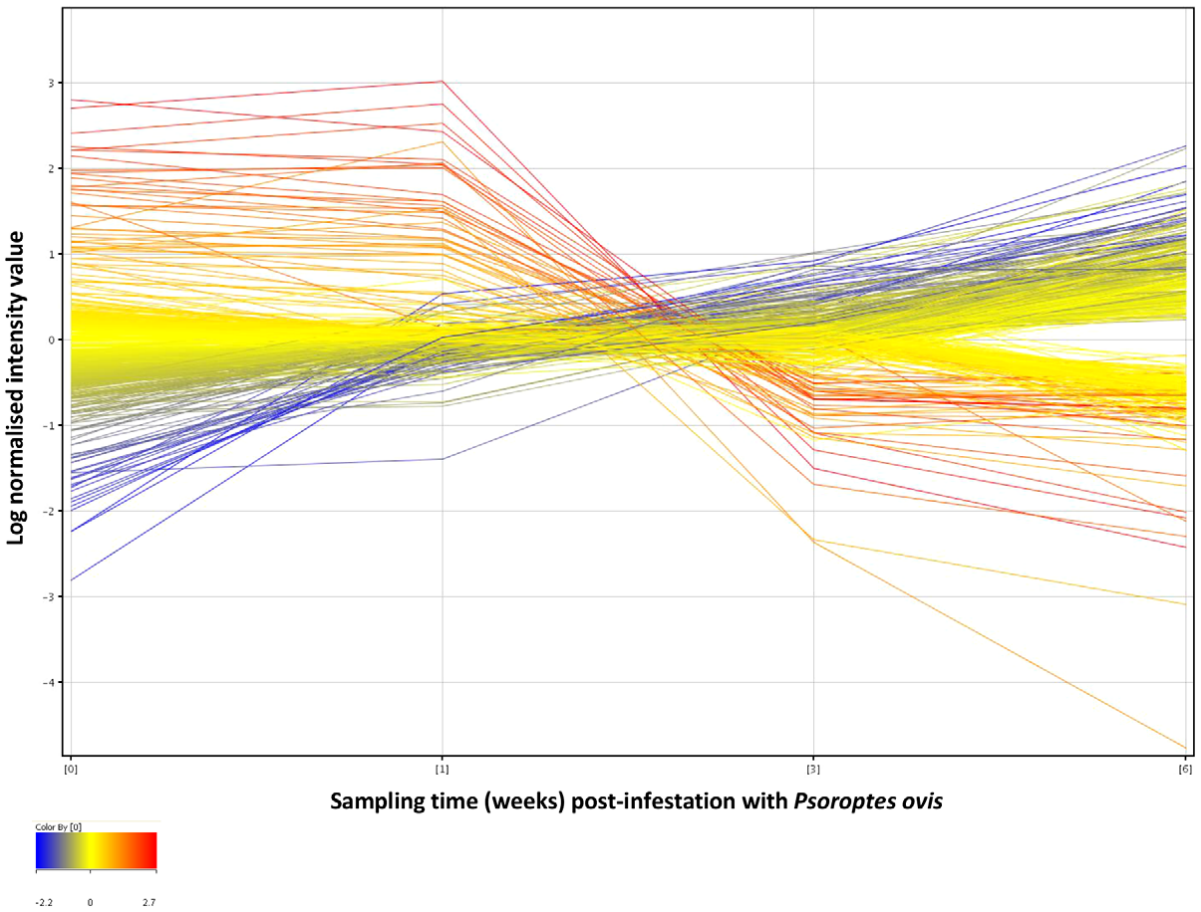
Profile plot of significantly differentially expressed genes in circulating leukocytes following infestation with *P*. *ovis*. Gene expression profiles for differentially expressed genes over the time course of infestation with *P. ovis*. Control (Time = 0), 1 week, 3 weeks and 6 weeks post infestation. Each line represents a single gene, colour coded by log normalised intensity values.

Using a one way ANOVA combined with a Student-Newman-Keuls (SNK) post-hoc test, 621 genes were designated as significantly differentially expressed with a fold change ≥1.8 in at least one of the 6 possible time point comparisons [non-infested control (C) *vs* 1 wpi, C *vs* 3wpi, C *vs* 6wpi, 1wpi *vs* 3wpi, 1wpi *vs* 6wpi and 3wpi *vs* 6wpi] (Figure 1). Gene symbol level annotation was available for 586 of the 621 probes (94%), and the relevant homologous human gene symbol was used where bovine annotation was unavailable. This annotation was obtained either from the Agilent bovine microarray annotation or from individual Basic Local Alignment Search tool (BLAST) analysis against the non-redundant (nr) nucleotide database of sequences represented by each probe, leaving 35 probes (6%) for which no annotation was available. Protocols of the experimental procedures, methods of analysis and microarray data are available as supplementary information in the European Bioinformatics Institute's ArrayExpress database (www.ebi.ac.uk/arrayexpress) under accession number E-TABM-1193.

### Network graph analysis and temporal clustering of host response data

**Figure 2:**
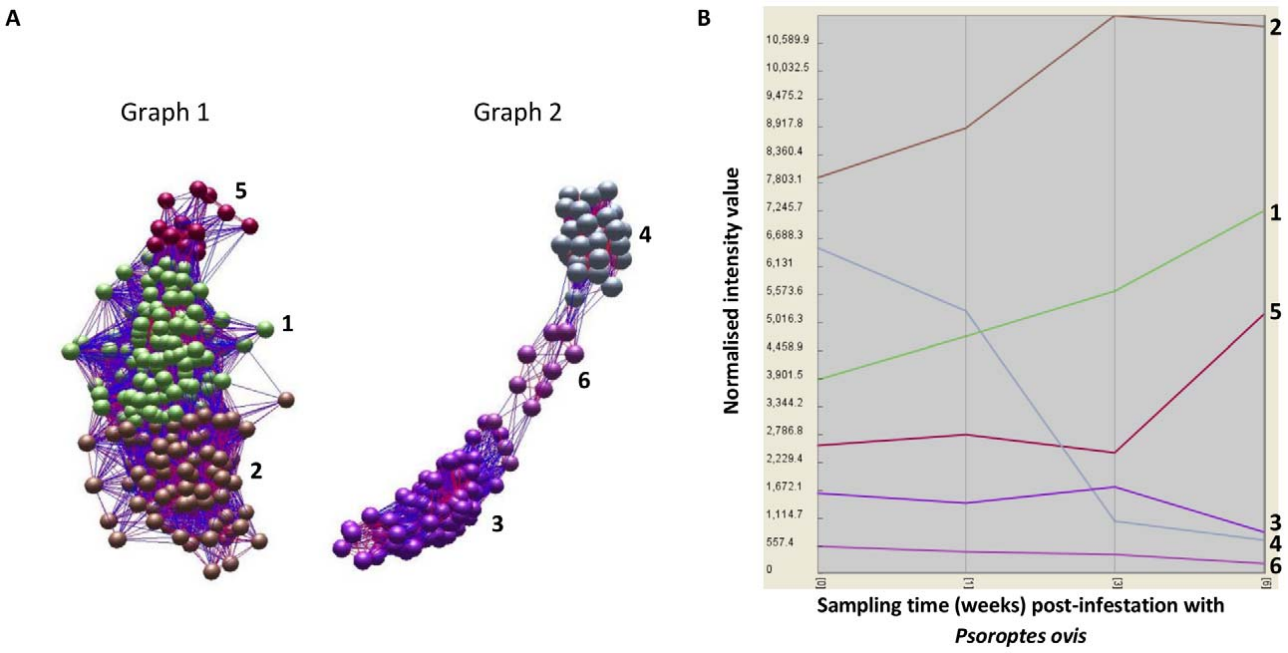
Network graph analysis and clustering of differentially expressed genes based on temporal expression profiles. **A**) Network graphs generated in BioLayout Express 3D utilising log-normalised expression data from 621 genes significantly differentially expressed in circulating leukocytes during time course of infestation with *P. ovis*. Graph 1 shows genes up-regulated with time and clustered into three distinct groups (Clusters 1, 2 and 5). Graph 2 shows genes down-regulated with time and clustered into three distinct groups (Clusters 3, 4 and 6). Each sphere (node) represents an individual gene and each line (edge) represents a relationship between nodes, i.e. correlated gene expression profile over time. **B**) Class mean expression profile plot showing mean expression profiles (based on the normalised mean intensity values for each gene within an individual cluster) for all genes in each of the six clusters.

The network visualisation and analysis tool BioLayout Express 3D was used to generate network graphs from the significantly differentially expressed genes. A Pearson correlation cut-off of ≥0.96 was applied, resulting in 617 of the 621 probes being incorporated into the graph, which consisted of 617 nodes and 44,168 edges. These data were used to generate the network graph, and an MCL inflation value of 2.1 was applied to cluster probes by their expression profiles across the time course of infestation with *P. ovis*. Five of the 617 probes failed to cluster at the selected MCL inflation value and were excluded from further analysis, leaving 612 probes. Two distinct sub-networks were identified within the network graph (Figure 2 a), each consisting of three gene clusters with distinct temporal patterns of expression over time. Graph 1 contained three clusters representing genes whose transcription levels were up-regulated over the time-course of infestation (Clusters 1, 2 and 5), whilst Graph 2 contained three clusters representing genes whose transcription levels were down-regulated with time (Clusters 3, 4 and 6); the details of each cluster are presented in Table 1. The genes clustered discretely based on their expression profiles across the time course of infestation (Figure 2 b). For example genes in Cluster 1 showed increased expression across the time course, peaking at 6 wpi. Genes in Cluster 2 showed peak expression by 3 wpi, which then began to decline by 6 wpi, whilst the expression levels of those in Cluster 5 increased moderately between 0–1 wpi, dropped between 1–3 wpi and then showed a much larger increase by 6 wpi. For the down-regulated clusters, Cluster 3 genes decreased slightly in their expression levels between 0–1 wpi, increased between 1–3 wpi before showing a pronounced decline in expression by 6 wpi. The expression of genes in Cluster 4 decreased across the time course with minimum expression at 6 wpi, whilst Cluster 6 genes showed a moderate but steady decline in expression from 0–6 wpi. In summary, the process of clustering based on gene expression profiles across the time course of infestation with *P. ovis* resulted in the formation of clear temporal clusters of genes. This process facilitated the downstream investigation of signalling pathways and gene networks in circulating leukocytes following exposure described in the following section.

**Table 1:** Network graph and clustering details for all differentially expressed genes.

Sub-network	Cluster ID	Regulation	Number of genes
**1**	1	Up	257
	2	Up	143
	5	Up	24
**2**	3	Down	140
	4	Down	39
	6	Down	9

Network analysis identified two distinct sub-networks within the network graph each representing 3 individual gene clusters with correlated expression profiles across the time course of infestation. Up/Down indicates direction of gene expression regulation following infestation with *P. ovis*. Of the 621 differentially expressed genes, 617 were included in the original network graph, of these 612 were incorporated into clusters 1–6.

### Pathway and gene network analysis of temporal host response to *P. ovis* infestation

To identify the temporal mechanisms and signalling pathways involved in the response of host circulating leukocytes following *P. ovis* infestation we undertook a pathway and gene network analysis of clusters 1–6, representing 612 of the 621 differentially expressed genes. The 10 genes with the highest fold-change (as compared to baseline expression at time = 0) from each of the clusters are detailed in Table 2 and the top five canonical signalling pathways from the IPA mapping are shown in Table 3. Table 4 shows the top gene networks associated with each cluster and these are discussed in more detail below. File S1 contains the fold-change, annotation and cluster assignment data for all of the significantly differentially expressed genes (p = ≤0.05) from the study. In summary, biological functions, canonical signalling pathways and gene networks associated with the individual temporal gene clusters were identified and these are described in further detail in the following sections.

**Table 2:** Top 10 fold-changing genes from each expression cluster.

Cluster ID	Gene Symbol	Gene Description	Fold Change*
***Cluster 1***	*MGAT3*	mannosyl (beta-1,4-)-glycoprotein beta-1,4-N-acetylglucosaminyltransferase	67.97
	*PDLIM1*	PDZ and LIM domain 1	52.51
	*TKDP3*	Trophoblast Kunitz domain protein 3	51.70
	*MYH3*	Myosin heavy chain 3	45.83
	*WWC1*	WW, C2 and coiled domain containing 1	31.36
	*C4orf19*	Chromosome 4 open reading frame 19	29.70
	*CEBPE*	CCAAT/enhancer binding protein (C/EBP) epsilon	23.09
	*TRPC6*	Transient receptor potential cation channel, subfamily C, member 6	22.49
	*EMR4P*	EGF-like module containing, mucin-like, hormone receptor-like 4	22.35
	*HOXB6*	Homeobox B6	22.29
***Cluster 2***	*COBRA1*	Co-factor of BRCA1	5.67
	*KIAA0649*	KIAA0649	3.72
	*MAPK7*	Mitogen-activated protein kinase 7	3.67
	*MYH1*	Myosin heavy chain 1	3.64
	*KCNQ1*	Potassium voltage-gated channel, KQT-like subfamily, member 1	3.42
	*FHL3*	Four and a half LIM domains 3	3.12
	*CTTN*	Cortactin	3.11
	*PNKD*	Paroxysmal nonkinesiogenic dyskinesia	3.07
	*MYH4*	Myosin heavy chain 4	2.96
	*RAB26*	RAB26, member RAS oncogene family	2.92
***Cluster 3***	*N/A*	N/A	−5.12
	*LDLRAD3*	Low density lipoprotein receptor class A domain containing 3	−4.24
	*N/A*	N/A	−4.08
	*AXL*	AXL receptor tyrosine kinase	−3.55
	*CSNK1G3*	Casein kinase 1, gamma 3	−3.14
	*N/A*	N/A	−3.05
	*C4orf16*	Chromosone 4 open reading frame 16	−3.05
	*ACSL3*	Acyl-CoA synthetase long chain family member 3	−3.04
	*KBTBD4*	Kelch repeat and BTB (POZ) domain containing 4	−2.86
	*HOOK1*	Hook homolog 1	−2.83
***Cluster 4***	*ALAS2*	Aminolevulinate, delta-, synthase 2	−59.17
	*AQP1*	Aquaporin 1	−25.83
	*SYNJ2*	Synaptojanin 2	−22.46
	*MAL*	Myelin and lymphocyte protein (T-lymphocyte maturation-associated protein)	−20.54
	*ZSWIM4*	Zinc finger, SWIM-type containing 4	−15.74
	*CYP4F12*	Cytochrome P450, family 4, subfamily F, polypeptide 12	−14.75
	*SLC22A1*	Solute carrier family 22 (organic cation transporter) member 1	−13.56
	*N/A*	N/A	−12.05
	*N/A*	N/A	−8.33
	*OR2S2*	Olfactory receptor, family 2, subfamily S, member 2	−7.84
***Cluster 5***	*SPP1*	Secreted phosphoprotein 1	53.34
	*PTX3*	Pentraxin-related gene, rapidly induced by IL1-beta	8.24
	*ANXA9*	Annexin A9	6.58
	*S100A9*	S100 calcium binding protein A9	6.45
	*S100A8*	S100 calcium binding protein A8	6.27
	*ECM1*	Extracellular matrix protein 1	6.26
	*IL1R2*	Interleukin 1 receptor, type II	5.55
	*KLHL4*	Kelch-like 4	5.21
	*LOC255649*	Oocyte-secreted protein variant 1	5.09
	*CDH4*	Cadherin 4, type 1, R-cadherin (retinal)	4.12
***Cluster 6***	*ZBTB41*	Zinc finger and BTB domain containing 41	−4.59
	*GBP3*	Guanylate binding protein 3	−4.53
	*C20orf194*	Chromosome 20 open reading frame 194	−3.79
	*MTRR*	5-methyltetrahydrofolate-homocyst cysteine methyltransferase reductase	−3.29
	*DMXL2*	DMX-like 2	−2.68
	*SF3B4*	Splicing factor 3b, subunit 4	−2.53
	*OSR2*	Odd-skipped related 2	−2.32
	*YAF2*	YY1 associated factor 2	−2.29
	*DEPDC7*	DEP domain containing 7	−2.06

* Fold change calculated based on the peak fold change (compared to baseline, time = 0) for each gene across the time course of infestation with *P. ovis*.

**Table 3:** Top 5 canonical signalling pathways associated with each gene expression cluster.

Cluster ID	Canonical Pathway	p-value*
***Cluster 1***	Chemokine signaling	1.7E-04
	G-protein coupled receptor signaling	1.06E-03
	CCR3 signalling in eosinophils	2.36E-03
	Pentose phosphate pathway	6.14E-03
	Nicotinate and nicotinamide metabolism	6.21E-03
***Cluster 2***	Calcium signaling	1.7E-04
	Gonadotrophin releasing hormone (GNRH) signaling	1.43E-03
	Integrin signaling	2.17E-03
	Tight junction signaling	4.01E-03
	Glycerophospholipid metabolism	7.94E-03
***Cluster 3***	DNA-double strand break repair by homologous recombination	1.62E-06
	Role of BRCA1 in DNA damage response	3.14E-05
	Hereditary breast cancer signaling	1.16E-04
	Purine metabolism	1.74E-03
	Role of NFAT in cardiac hypertrophy	6.48E-03
***Cluster 4***	Metabolism of xenobiotics by Cytochrome P450	1.69-02
	Fatty acid metabolism	1.89E-02
	Tryptophan metabolism	2.1E-02
	Extrinsic prothrombin activation pathway	2.75E-02
	Ascorbate and aldarate metabolism	3.09E-02
***Cluster 5***	Role of IL17A in psoriasis	1.51E-04
	Hepatic fibrosis/hepatic stellate cell activation	1E-03
	NF-kB signaling	1.67E-03
	Role of osteoblasts, osteoclasts and chondrocytes in rheumatoid arthritis	3.59E-03
	Growth hormone signaling	4.2E-03
***Cluster 6***	Systemic lupus erythematosus signaling	5.92E-02

* p-value calculated using Fisher's exact test determining the probability that association between genes in the data set and canonical pathway is due to chance alone.

**Table 4:** Top gene networks and associated biological functions for gene expression clusters.

Cluster ID	Network ID	Associated Gene Network Functions	Score*
***Cluster 1***	1	Cellular development, cellular growth and proliferation	46
	2	Inflammatory response, cell signalling and inflammatory disease	38
	3	Lipid metabolism, small molecule biochemistry and ophthalmic disease	38
	4	Connective tissue disorders, dental disease, developmental disorder	33
	5	Connective tissue disorders, genetic disorder, skeletal and muscular disorders	28
***Cluster 2***	1	Cellular assembly and organisation, cellular function and maintenance	46
	2	Cellular assembly and organization, skeletal and muscular system development and function	43
	3	Cellular development, cellular growth and proliferation, connective tissue development and function	22
	4	Cardiovascular system development and function, organ development, molecular transport	22
	5	Cardiovascular disease, cell death, gastrointestinal disease	22
***Cluster 3***	1	Lipid metabolism, small molecule biochemistry, molecular transport	41
	2	Cell cycle, DNA replication, recombination and repair, cellular assembly and organization	33
	3	Connective tissue disorders, genetic disorder, ophthalmic disease	26
	4	Developmental disorder, genetic disorder, neurological disease	22
	5	RNA post-translational modification, cell cycle, haematological system development and function	22
***Cluster 4***	1	Cellular development, inflammatory response, cellular growth and proliferation	33
	2	Gene expression, cardiovascular system development and function, cell morphology	31
***Cluster 5***	1	Cellular movement, haematopoiesis, immune cell trafficking	33
	2	Cardiovascular system development and function, organismal development	20
***Cluster 6***	1	Cellular growth and proliferation, cellular development, haematological system development	14

* Gene network score is a numerical value used to rank networks according to how relevant they are to the genes in the input dataset. IPA uses a right-tailed Fisher's test to calculate the p-value for networks. A score of 10 indicates a p = 10^−10^ chance that genes in that network are associated solely by chance. Only gene networks with a score >10 shown.

### Biological functions, canonical signalling pathways and gene networks associated with clusters of genes up-regulated following infestation with *P. ovis*

#### Cluster 1

**Figure 3:**
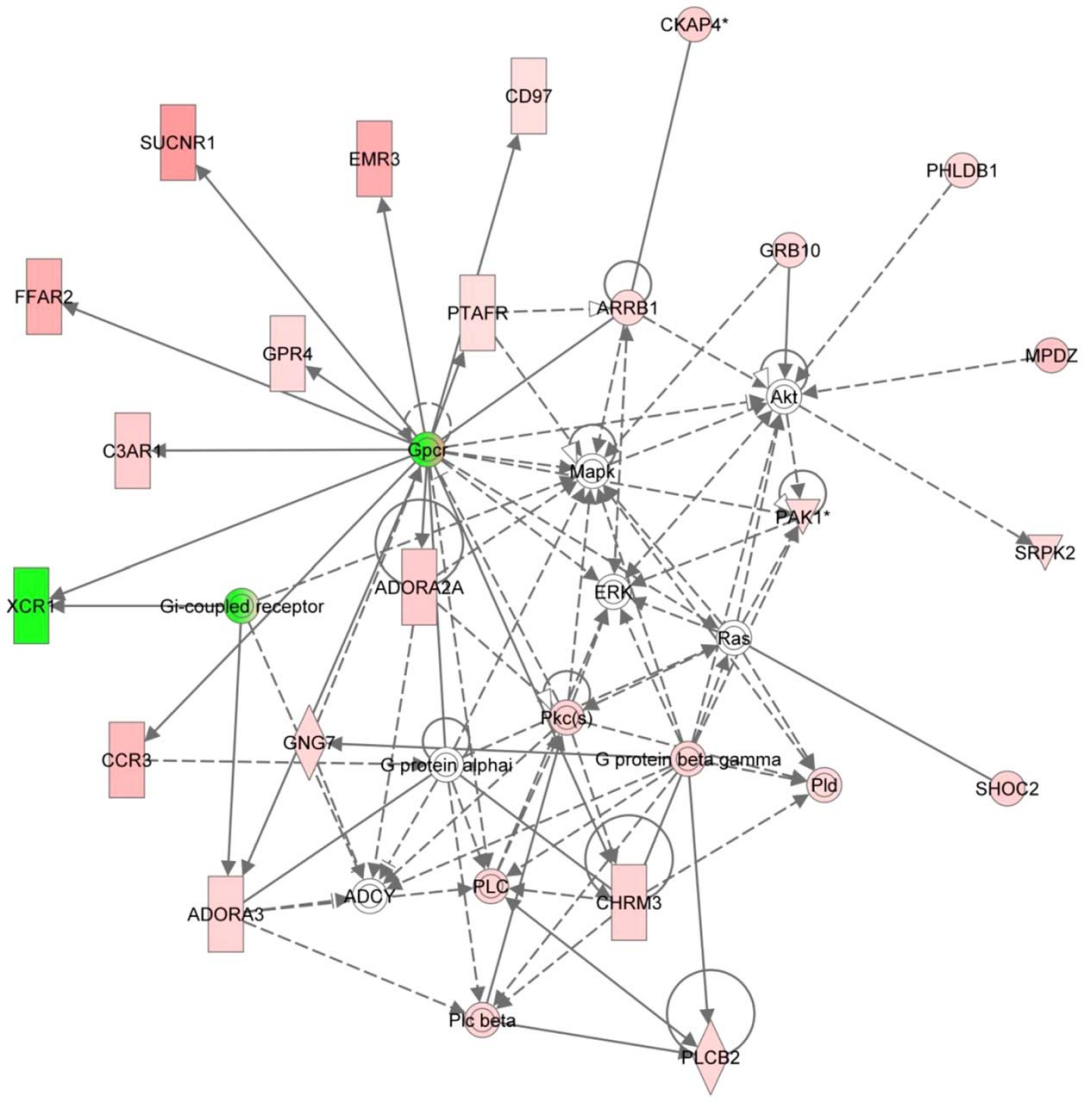
Graphical representation of genes in Cluster 1, Network 2 showing up-regulated expression across the 6 week time course, highlighting the involvement of the pro-inflammatory mediators *CCR3* and *C3AR1* in the host inflammatory response to *P. ovis* infestation. Key: Individual nodes represent proteins with relationships represented by edges. Nodes coloured by gene expression, red indicating up-regulation, green indicating down-regulation and white indicating gene/factor not differentially expressed but with defined relationship to other genes in network. Arrows indicate directional relationships.

The expression profile of genes within this cluster showed a gradual increase over the time course of infestation. Cluster 1 consisted of 257 genes and biological functions associated with these included “Infectious disease” (35 genes, p = 1.68E^−08^−1.17E^−02^), “Respiratory disease” (26 genes, p = 1.68E^−08^−1.45E^−02^) and “Inflammatory response” (37 genes, p = 4.86E^−06^−2.33E^−02^), whilst the top canonical pathways represented were “Chemokine signalling”, “G-protein coupled receptor signalling” and “CCR3 signalling in eosinophils” (Table 3). The two highest scoring gene networks represented in Cluster 1 showed enrichment for genes involved in cellular development, and inflammatory response (Table 4). This cluster contained a number of complement components, including complement factor 4 binding protein-alpha [*C4BPA* (6.5-fold up by 6 wpi), *C4BPB* (12-fold up by 6 wpi), complement component 3a receptor 1[*C3AR1* (6.3-fold up by 6 wpi)] and complement factor properdin (*CFP* (3-fold up by 6 wpi)]. An exemplar network from this cluster is shown in Figure 3 (Cluster 1, Network 2, also see Table 4). This network was enriched for the following biological functions, inflammatory response, cell signalling and inflammatory disease and highlighted the up-regulation at the transcription level of a number of genes involved in the instigation of the inflammatory response, including *CCR3* (7.6-fold up by 6 wpi) and *C3AR1* (6.3-fold up by 6 wpi). As such Cluster 1 showed enrichment for a number of factors involved in the innate immune response. In particular, genes involved in the instigation and development of a pro-inflammatory response were enriched within this cluster, including roles for eosinophil recruitment and complement activation, the expression of which increased as disease signs and the subsequent inflammatory response intensified.

#### Cluster 2

**Figure 4:**
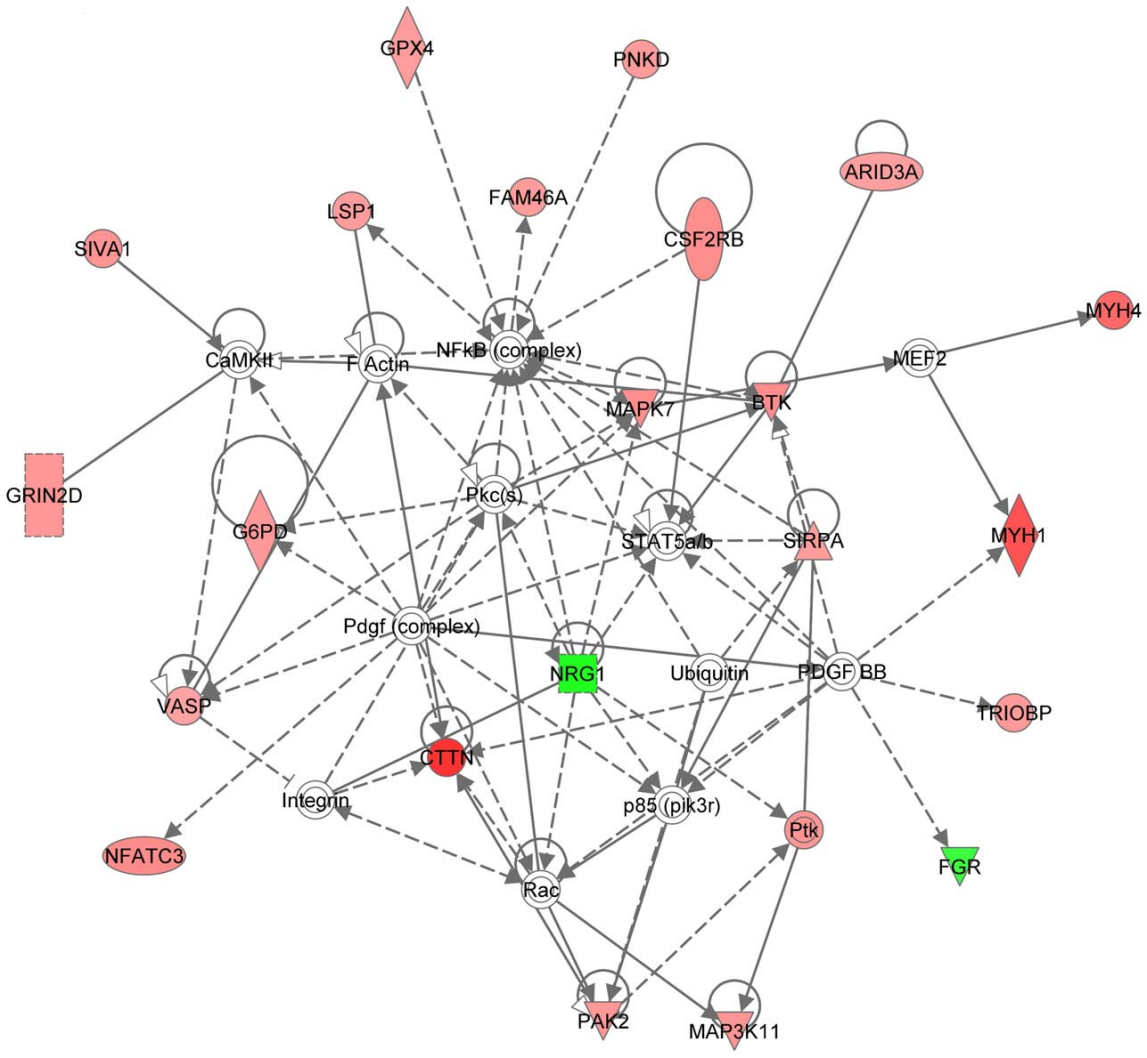
Graphical representation of genes within Cluster 2, Network 2, showing up-regulated expression by 3 wpi, which then declined by 6 wpi. This network highlights the role of increased leukocyte extravasation evidenced by the up-regulation of key integrins and adhesion molecules. NB: Integrin genes *ITGAE* and *ITGA2B* marked by the circle titled Integrins. Key: Individual nodes represent proteins with relationships represented by edges. Nodes coloured by gene expression, red indicating up-regulation, green indicating down-regulation and white indicating gene/factor not differentially expressed but with defined relationship to other genes in network. Arrows indicate directional relationships.

The genes within this cluster showed an initial increase in expression levels which then began to decline in magnitude between 3–6 weeks post-infestation. Cluster 2 contained 143 genes and the top biological functions represented were “Infectious disease” (26 genes, p = 1.24E^−04^−2.55E^−02^), “Inflammatory response” (23 genes, p = 2.37E^−04^−2.12E^−02^) and “Respiratory disease” (13 genes, p = 8.45E^−04^−2E^−02^), with the top canonical pathways being “Calcium signalling”, “Gonadotrophin releasing hormone (GNRH) signalling”, “Integrin signalling” and “Tight junction signalling” (Table 3). Networks represented within Cluster 2 were enriched for genes associated with cellular assembly and organisation, cellular function and maintenance and skeletal and muscular system development. In particular, Networks 1 and 2 [Figure 4 (Showing Cluster 2, Network 2) and Table 4] contain a number of genes involved in cell migration and adhesion; integrin alpha E [*CD103* (*ITGAE*) (2.1-fold up regulated by 6 wpi)], *ITGA2B* (2.2-fold up by 6 wpi) shown by the group icon ‘Integrin’ in Figure 4, lymphocyte specific protein 1 [*LSP1* (1.9-fold by 6 wpi)], *ICAM3* (2.9-fold up by 6 wpi), cortactin [*CTTN* (3.1-fold up by 6 wpi)], signal regulatory protein alpha [*SIRPA* (2.1-fold up by 6 wpi)], drebrin 1 [*DBN1* (2.3-fold up by 6 wpi)] and calponin 2 [*CNN2* (1.8-fold up by 6 wpi)]. Cluster 2 also contained the gene encoding the plasminogen activator, urokinase (*PLAU*, 6.3-fold by 6 wpi) which has been shown to expressed by activated leukocytes. Therefore, Cluster 2 showed enrichment for a number of factors induced during the pro-inflammatory response with roles in the activation of leukocytes and triggering their subsequent extravasation into the site of tissue inflammation. The expression of these genes were up-regulated early on in the infestation, followed by a subsequent decline between 3 and 6 wpi.

#### Cluster 5

Genes within this cluster showed a distinct increase in expression levels between 3 and 6 weeks pi. Cluster 5 consisted of 24 genes and the top biological functions associated with these genes included “Cancer” (17 genes, p = 2.42E^−07^−1.7E^−02^), “Reproductive system disease” (13 genes, p = 8.07E^−07^−1.28E^−02^) and “Inflammatory response” (9 genes, p = 1E^−06^−1.7E^−02^). The top canonical signalling pathways were “Role of IL-17A in Psoriasis”, “Hepatic fibrosis” and “NF-kB signalling” (Table 3). Network 1 from Cluster 5 showed enrichment for factors associated with cellular movement and immune cell trafficking, including the receptor for interleukin 1 (*IL1A*/*IL1B*), interleukin 1 receptor 2 (*IL1R2*), secreted phosphoprotein 1 (*SPP1*, 53-fold up by 6 wpi), the S100 calcium binding proteins *S100A8* (6.3-fold up by 6 wpi) and *S100A9* (6.5-fold up by 6 wpi), pentraxin 3 [*PTX3* (8.2-fold up by 6 wpi)] and the selectin P ligand [*SELPLG* (1.8-fold up by 6wpi)] (Table 4). Similar to the other up-regulated gene clusters (i.e. Clusters 1 and 2), Cluster 5 was also enriched for genes involved in the instigation and maintenance of the inflammatory response. The up-regulation of the selectin P ligand gene, *SELPLG*, within this cluster may be indicative of increased leukocyte activation and extravasation and could be related to the expanding skin lesion and worsening pathology at this time. This may also be reflected by the increased expression of a number of genes involved in wound healing and immune cell trafficking at this time, i.e. *PTX3* and *SPP1*.

### Biological functions, canonical signalling pathways and gene networks associated with clusters of genes down-regulated following infestation with *P. ovis*

#### Cluster 3

The major feature of gene expression within Cluster 3 was down-regulation of transcript levels between 3–6 weeks pi. This cluster contained 140 genes and the biological functions associated with these were “Developmental disorder” (15 genes, p = 1.16E^−04^−3.85E^−02^), “Genetic disorder” (43 genes, p = 1.16E^−04^−4.48E^−02^), “Neurological disease” (9 genes, p = 1.16E^−04^−3.22E^−02^) and “Gastrointestinal disease” (42 genes, p = 8.66E^−04^−4.48E^−02^). Top signalling pathways were “DNA double-strand break repair by homologous recombination”, “Role of BRCA1 in DNA damage response” and “Hereditary breast cancer signalling” (Table 3). The top scoring network from Cluster 3 showed enrichment for genes involved in lipid metabolism, small molecule biochemistry and molecular transport (Table 4). This network demonstrated the differential expression of the receptor for oncostatin-M (*OSM*) *OSMR* (FC = −1.8 by 6 wpi, compared to baseline), which regulates the production of pro-inflammatory cytokines [i.e. interleukin-6 (*IL6*), colony stimulating factor 2 (*CSF2*) and *CSF3* [20]]. This network also contained indoleamine 2,3-dioxygenase 1 [*IDO1* (FC = −3.2 by 6 wpi compared to baseline)] which has roles in immune defence and antioxidant activity [21]. In a previous study analysing transcriptional changes in sheep skin following infestation with *P. ovis*, the expression of *CSF2*, also known as granulocyte macrophage colony stimulating factor (*GM-*CSF) was found to be significantly increased within 1 hour of exposure [6]. CSF2 stimulates the growth and differentiation of granulocytes, macrophages and eosinophils [22]. Cluster 2 contained the gene encoding the CSF2 receptor, *CSF2RB* which showed 2.9-fold up-regulation in expression levels by 6 wpi in circulating leukocytes in the current study and may be indicative of these cells responding to CSF2 expressed at the site of infestation. Therefore, Cluster 3, which showed peak down-regulation between 3 and 6 wpi was enriched for genes involved in the innate immune response with a focus on down-regulation of a number of pro-inflammatory pathways.

#### Cluster 4

**Figure 5:**
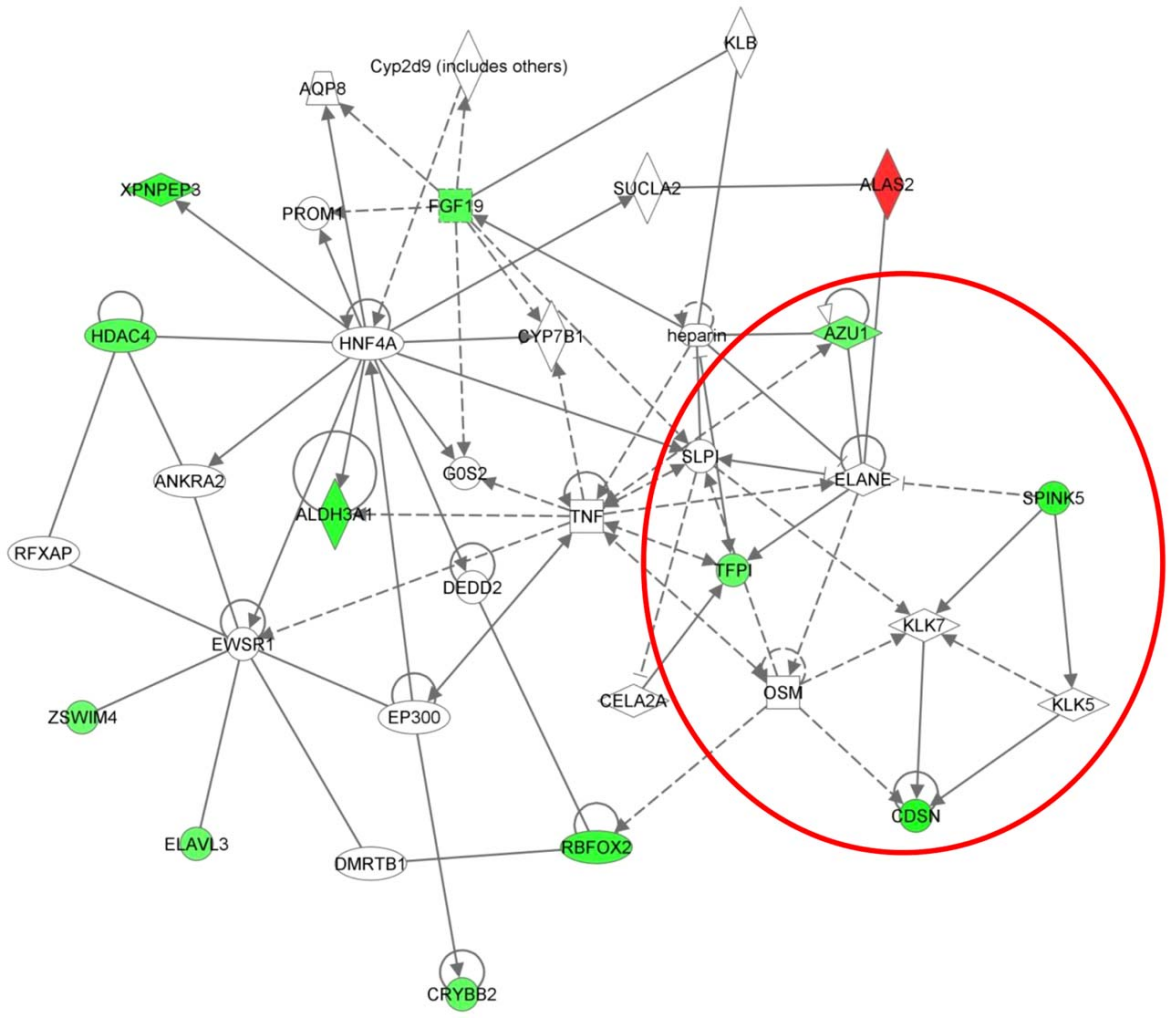
Graphical representation of genes within Cluster 4, Network 2, showing down-regulated expression across the 6 week time course. Genes within the red circle are members of a sub-set within the network with defined roles in skin barrier function and are described further in the main text. Key: Individual nodes represent proteins with relationships represented by edges. Nodes coloured by gene expression, red indicating up-regulation, green indicating down-regulation and white indicating gene/factor not differentially expressed but with defined relationship to other genes in network. Arrows indicate directional relationships.

The expression of genes in Cluster 4 was reduced between 0 and 1 wpi (Figure 2 b). This reduction was accentuated between weeks 1 and 3 post-infestation and expression levels remained low and declining between weeks 3 and 6 pi. Cluster 4 consisted of 39 genes and the top biological functions associated with these genes were “Dermatological disease and conditions” (2 genes, p = 6.09E^−05^−1.74E^−03^), “Genetic disorder” (20 genes, p = 6.09E^−05^−2.92E^−02^) and “Inflammatory disorder” (7 genes, p = 2E^−04^−3.96E^−02^), whilst the top signalling pathways were “Metabolism of xenobiotics by Cytochrome P450”, “Fatty acid metabolism” and “Tryptophan metabolism” (Table 3). Two significant gene networks were identified from Cluster 4 (Networks 1 and 2) and were associated with genes involved in cellular development, inflammatory response, cellular growth and proliferation, gene expression and cell morphology (Table 4). Network 2 contained a sub-set of genes (Figure 5) which included the serine peptidase inhibitor, kazal type 5 (*SPINK5*), tissue factor pathway inhibitor (*TFPI*), azurocidin 1(*AZU1*), corneodesmosin (*CDSN*), *OSM*, neutrophil elastase (*ELANE*), secretory leukocyte peptidase inhibitor (*SLPI*) and kalikrein-5 (*KLK5*) and -7 (*KLK7*). Of these, *SPINK5*, *CDSN*, *TFPI* and *AZU1* were differentially expressed in circulating leukocytes following infestation with *P. ovis*, whilst the remaining genes were associated with these genes within the network but were either not differentially expressed or not present on the array. Re-analysis of this sub-set within IPA generated a gene network enriched for the biological function of “Dermatological diseases and conditions”. *KLK5*, *KLK7*, *SPINK5*, *CDSN* and *SLPI* are implicated in the regulation of skin barrier function *via* their roles in the kallikrein-kinin pathway. Therefore, Cluster 4 showed enrichment for genes involved in the maintenance of skin barrier function, which were down-regulated across the time course of infestation with a pronounced decline between 1 and 3 wpi.

#### Cluster 6

The genes in this cluster showed a moderate down-regulation in their expression between 3 and 6 weeks pi. Only nine genes were represented in this cluster leaving insufficient data for effective pathway mapping. One signalling pathway was associated with these genes “Systemic lupus erythematosus signalling” (Table 3). However, no significant networks were associated with this cluster.

### qPCR validation of microarray data

qPCR confirmation of the microarray results was undertaken for 10 putatively differentially expressed genes. qPCR assays were based on bovine transcripts and performed with ovine circulating leukocyte cDNA generated from the same RNA samples used for the microarray study. Overall the fold change data showed a mean correlation co-efficient of 0.82 between the microarray and qPCR datasets (Data not shown). The differential expression of the selected genes was considered to be validated as the qPCR expression profile over the time course of infestation for the majority of these genes mirrored that of the microarray data.

## Discussion

This study had two main aims: i) to examine the systemic inflammatory response in a production animal using an economically-important host:pathogen model and, ii) to establish whether circulating leukocytes in sheep could act as remote ‘sentinels’ of the cutaneous inflammatory response characteristic of infestation with *P. ovis*. To achieve these aims we investigated the whole population of circulating leukocytes over a time course of infestation rather than fractionating samples into distinct cell sub-sets prior to analysis. This approach was followed to give a global picture of gene expression profiles in circulating leucocytes but also allowed us to avoid a prolonged period between sampling blood and the subsequent RNA stabilisation and extraction, which can lead to significant *ex vivo* changes in gene expression profiles [23]. In addition, by fractionating and isolating leukocytes from whole blood prior to RNA extraction we were able to limit contamination with globin transcripts, which can represent up to 70% of total mRNA and lead to significant skewing of gene expression data [24], [25]. One limitation of this approach is that, as blood is a relatively heterogenous tissue comprised of multiple cell types and sub-sets, it does not allow discrimination between differential gene expression due to changes in cell sub-set proportions over time and changes in sub-set specific gene expression. As such, increased gene expression may equally represent an increase in the number of a particular cell sub-set in circulation, i.e. neutrophils, or increased expression by individual cells. The use of differential cell counts may have aided in further elucidation of these responses, in particular with cell sub-set specific expression patterns, i.e. *CCR3* in eosinophils and basophils, but the main focus here was the elucidation of expression changes in the whole leukocyte population as a ‘sentinel’ of local tissue events. In addition, as the clustering analysis highlighted the presence of a range of different gene expression patterns across the time course of infestation it is unlikely that all of these were attributable to cell sub-set specific expression/expansion. At the instigation of this study no suitable ovine microarray platforms were available; as such we opted to perform a cross-species microarray analysis using the Agilent bovine gene expression microarray. Cattle and sheep orthologues share a mean transcript sequence identity of 97% [26], [27], [28] and previous studies have successfully used bovine microarrays for the analysis of ovine samples, further supporting the use of this type of analysis [29], [30], [31].

Infestation of sheep with *P. ovis* results in a cutaneous pro-inflammatory response within minutes of contact [6]. However, the timing and nature of the systemic inflammatory response is unknown so we conducted our analyses over a 6 week time course of infestation, during which the characteristic skin lesion develops. The process of clustering genes based on their expression profiles following infestation represented a successful method of grouping genes and facilitated further investigation of the signalling events in circulating leukocytes following exposure. A clearer picture is now emerging of the molecular events in these circulating cells following infestation, characterised by the up-regulated expression of genes involved in a number of biological pathways including complement activation, chemokine signalling, integrin and tight junction signalling and calcium activation. In addition, repressed pathways included those involved in the regulation of skin barrier function and certain aspects of the inflammatory response which occur later in the course of infestation. These events and pathways are discussed in further detail below in the context of their temporal regulation during infestation with *P. ovis*.

### Pro-inflammatory response factors

One of the main implications of the local pro-inflammatory cascade following infestation with *P. ovis* is activation and recruitment of circulating leukocytes. Those found to be prominent at the site of infestation within the first few hours post-infestation are neutrophils and eosinophils [5], for which IL8 represents a powerful chemoattractant [32]. These cells react to the presence of pro-inflammatory cytokines through the expression of receptors [33] and, of particular interest in the present study because of their roles in the activation of immune cells, were the increased expression of *CCR3* and *C3AR1* within Cluster 1, and the *IL4* receptor (*IL4R*, 1.8-fold up by 6 wpi) within Cluster 2. CCR3 acts as a receptor for a range of chemokines including, chemokine (C-C motif) ligand 11 (CCL11 or eotaxin), (CCL26 (eotaxin-3), CCL5 (RANTES), CCL7 [Monocyte chemoattractant protein 3 (MCP-3)] and CCL13 (MCP-4), a number of which have previously been shown to be up-regulated in sheep skin within a few hours of exposure to *P. ovis* [6]. C3AR1 is a receptor for the pro-inflammatory anaphylatoxin C3a and stimulates chemotaxis, mast cell degranulation and production of superoxide anions [34]. *CCR3* expression was also shown previously to be increased in sheep skin following infestation with *P. ovis* and may be indicative of eosinophil and/or basophil activation (*CCR3*) and also complement activation (*C3AR1*) in response to infestation [6], [35]. In addition, the increased expression levels of *IL4R* in circulating leukocytes may indicate immune cell responses to the presence of locally produced *IL4* and *IL13*, both of which play prominent roles in the development of a pro-allergic, Th2 biased, immune response and have also been shown to be up-regulated in *P. ovis* infested sheep skin [6], [36], [37]. We have demonstrated that a number of pro-inflammatory genes and signalling pathways are involved during the early stages of a *P. ovis* infestation. The temporal nature of this response was reflected by the enrichment of these pathways across both Clusters 1 and 2, representing up-regulated gene expression between 0–1 and 1–3 wpi, respectively.

### Leukocyte extravasation and activation

Once activated, immune mediator cells express ligands and receptors which aid binding to the endothelial lining, initiating leukocyte extravasation and immune cell infiltration [38], [39], [40], [41]. We have demonstrated previously the increased expression of transcripts representing selectins, integrins and other adhesion molecules including selectin-E (*SELE*), -P (*SELP*), -L (*SELL*), integrin beta 2 (*ITGB2*), *ITGB6*, intercellular adhesion molecule 1 (*ICAM1*) and *ICAM3* in sheep skin following infestation with *P. ovis* [6]. In the present study, analysing gene expression in circulating leukocytes in response to *P. ovis* infestation, Cluster 2 was enriched for genes involved in the canonical pathways “Integrin signalling” and “Tight junction signalling” and amongst the up-regulated genes in this cluster were those encoding *ITGAE* and *ITGA2B*, *LSP1* and *ICAM3*, which acts as a ligand for ITGB2; also previously shown to be up-regulated in *P. ovis* infested sheep skin [6], [42]. In Cluster 5, increased expression of *SELPLG* was observed, which acts as a receptor for the selectin molecules (SELE, SELP and SELL) expressed on the endothelial surface during an inflammatory reaction [43]. This receptor-ligand interaction is critical in the initial capture of circulating leukocytes and *SELPLG* also mediates the rolling of leukocytes, preparing these cells for extravasation into inflamed tissue [43]. Once activated circulating leukocytes bind to specific ligands/receptors on epithelial cells, slowing their progress and encouraging the leukocytes to begin rolling along the epithelial surface. At this point their morphology changes and the cells become flattened in preparation for the process of extravasating across the epithelial surface to the site of inflamed tissue. This process is reflected in a temporal manner with enrichment for genes involved in leukocyte activation and extravasation across both Clusters 2 and 5, representing up-regulated gene expression between 1–3 and 3–6 wpi.

### Pro-inflammatory mediator release

In addition to triggering cell extravasation, the activation of immune mediator cells also acts as a signal for the release of pro-inflammatory molecules and Cluster 5 contained a number of key pro-inflammatory mediators, i.e. *PTX3*, *S100A8*, *S100A9*, *SPP1* and myeloperoxidase (*MPO*) which forms the major component of neutrophil azurophilic granules [44] (2.7-fold up between 3–6 wpi). Together, S100A8 and S100A9 represent up to 60% of the cytosolic protein in neutrophils and are also released by keratinocytes [45], [46], [47]. Previous analysis of the skin response to *P. ovis* infestation demonstrated an increase in the expression of *S100A9* [6] and the putative conserved roles of S100A8 and S100A9 in contact dermatitis [48] and sheep scab have been discussed previously [6]. Importantly, S100A8 and S100A9 have been shown to regulate vascular inflammation and to promote recruitment of leukocytes to local tissue sites [49]. The release of these factors by activated keratinocytes has been implicated in the development of skin disease and may initiate immune mediator invasion, which could be exacerbated further by release from circulating neutrophils as implied from the analysis described herein [47]. SPP1 has roles in the migration and recruitment of neutrophils and has been implicated in the development of allergic contact dermatitis - as such it may also play an important role in sheep scab pathogenesis [50].

### Skin barrier disruption

A sub-set was identified within Cluster 4, network 2, enriched for genes involved in “Dermatological diseases and conditions”. This sub-set included a number of genes including *KLK5*, *KLK7*, *SPINK5*, *CDSN* and *SLPI* with decreased expression levels following infestation and with functions in the regulation of skin barrier function. The protease inhibitors *SPINK5* and *SLPI* have anti-inflammatory roles that contribute to the integrity of the skin barrier by protecting epithelial surfaces from the action of pro-inflammatory proteases [51], [52]. The maintenance of an effective skin barrier plays a crucial role in the prevention of allergen sensitisation and previous analysis of the skin response to *P. ovis* infestation demonstrated the down-regulation of a number of genes involved in the process of epidermal differentiation, i.e. filaggrin, loricrin and involucrin [6]. It has also been demonstrated that a Th2 response, particularly involving the increased expression of *IL4* and *IL13*, as demonstrated in sheep scab infestation [6], can affect skin barrier integrity by down-regulating expression of these genes [53], [54].

### Complement system and systemic inflammatory response

The complement system forms an integral arm of innate immunity and plays a crucial role in targeting pathogens and other foreign entities through opsonisation, cell lysis and immune cell recruitment [55]. Activation of complement has previously been described in the host response to *P. ovis* in sheep and in the response of humans to the scabies mite *Sarcoptes scabiei* and house dust mite (HDM) allergens [6], [56], [57]. Complement factor I (*CFI*) present in Cluster 5, displayed increased levels of expression following infestation. In addition, Cluster 1 contained a number of up-regulated complement factor genes, including *C4BPA*, *C4BPB*, *C3AR1* and *CFP*. C4BPA and C4BPB control activation of the classical complement pathway, whilst C3AR1 is the receptor for complement component 3a (C3a), produced from C3 (along with C3b) following proteolytic cleavage by C3 convertase [58]. Taken together these findings suggest activation of complement at the systemic level, in circulating leukocytes, following infestation with *P. ovis*, the outcome of which is likely to be the targeting of mites and/or mite antigens by the host immune response. Previous analysis of the cutaneous response to infestation with *P. ovis* demonstrated increased expression levels of *CFP*, *CFI*, *CFB*, *C4BPA* and also *C5AR1* [6]. In addition, previous studies have shown that the HDM allergen Der p 3 [for which a *P. ovis* homologue, Pso o 3 exists, (S.T.G. Burgess, Unpublished data)] has been shown to be capable of cleaving both C3 and C5, producing the anaphylatoxins C3a and C5a, respectively [56]. These factors trigger the release of histamine from mast cells and also act as chemoattractants for circulating leukocytes, i.e. neutrophils and their release may therefore further contribute to the pathogenesis of inflammatory and allergic disease [56]. Studies involving *S. scabiei* have shown that these mites produce a repertoire of inactivated serine proteases termed scabies mite inactivated protease paralogs (SMIPPs), which inhibit complement, protecting mites from complement-mediated damage [57]. To date, no *P. ovis* homologues of the *S. scabiei* SMIPPs have been identified, although *P. ovis* genomic and transcriptomic sequence data remains limited. A second mechanism through which complement may be activated in response to *P. ovis* is via the clotting cascade following local tissue injury [59]. This cascade produces the vasoactive peptides killidin and bradykinin which increase vascular permeability at the site of injury [59]. The HDM serine protease Der p 3 has been shown to activate this cascade, resulting in the generation of bradykinin and instigation of an inflammatory response [60], [61]. Cluster 2 also contained *PLAU* which is expressed by activated leukocytes and acts to enhance cell adhesion [62]. PLAU cleaves plasminogen, releasing plasmin and triggering the process of fibrinolysis, which acts as a counter balance to the clotting cascade by lysing fibrin and preventing blood clot formation [63]. Analysis of *P. ovis* protease activity demonstrated that mite extracts were capable of digesting fibronectin, indicating a possible role for the fibronectin pathway in lesion development [64].

Based on the observations herein we propose that contact with sheep scab mite allergens may trigger the release of pro-inflammatory cytokines and activation of the complement pathway, these factors then act as chemotactic agents for the recruitment of neutrophils to the site of infestation and the induction of an acute phase response. In addition, the anaphylatoxins C3a and C5a, either produced locally by complement activation, or following cleavage by mite derived proteases, induce local mast cell degranulation and release of histamine, leading to vasodilation and contraction of smooth muscle. The subsequent increase in vascular permeability and leukocyte extravasation, triggered by cytokine activated epithelium and leukocyte activation induces further inflammation at the site of infestation, leading to the worsening of disease symptoms, i.e. the inflamed skin and exudate characteristic of the sheep scab lesion. Here we used circulating leukocyte responses to assess the systemic effects of *P. ovis* infestation. Whilst this analysis has provided key insights into the broader inflammatory response to infestation, these cells have also acted as a proxy of events occurring in the skin, providing novel insights into the mechanisms by which a local allergen-induced inflammatory response may be controlled.

## Supporting Information

File S1 **Contains the fold-change, annotation and cluster assignment data for all of the significantly differentially expressed genes (p = ≤0.05) in circulating leukocyte RNA following infestation with** ***P. ovis*** **.**
(XLSX)
